# Da-Cheng-Qi decoction improves severe acute pancreatitis-associated acute lung injury by interfering with intestinal lymphatic pathway and reducing HMGB1-induced inflammatory response in rats

**DOI:** 10.1080/13880209.2022.2160768

**Published:** 2023-01-09

**Authors:** Xiaowen Liu, Lin Yuan, Yishuang Tang, Yue Wu, Jing Kong, Bingduo Zhou, Xiaosu Wang, Min Lin, Yading Li, Gaofan Xu, Yi Wang, Tingting Xu, Cong He, Shengquan Fang, Shengliang Zhu

**Affiliations:** a Department of Gastroenterology, Yueyang Hospital of Integrated Traditional Chinese and Western Medicine, Shanghai University of Traditional Chinese Medicine, Shanghai, China; b Experiment Center for Science and Technology, Shanghai University of Traditional Chinese Medicine, Shanghai, China

**Keywords:** Intestinal barrier function, traditional Chinese medicine, NF-κB p65

## Abstract

**Context:**

Da-Cheng-Qi Decoction (DCQD) has a significant effect on Severe Acute Pancreatitis-Associated Acute Lung Injury (SAP-ALI).

**Objective:**

To explore the mechanism of DCQD in the treatment of SAP-ALI based on intestinal barrier function and intestinal lymphatic pathway.

**Materials and methods:**

Forty-five Sprague-Dawley rats were divided into three groups: sham operation, model, and DCQD. The SAP model was induced by a retrograde infusion of 5.0% sodium taurocholate solution (1 mg/kg) at a constant rate of 12 mL/h using an infusion pump into the bile-pancreatic duct. Sham operation and model group were given 0.9% normal saline, while DCQD group was given DCQD (5.99 g/kg/d) by gavage 1 h before operation and 1, 11 and 23 h after operation. The levels of HMGB1, RAGE, TNF-α, IL-6, ICAM-1, d-LA, DAO in blood and MPO in lung were detected using ELISA. The expression of HMGB1, RAGE, NF-κB p65 in mesenteric lymph nodes and lung were determined.

**Results:**

Compared with SAP group, DCQD significantly reduced the histopathological scoring of pancreatic tissue (SAP, 2.80 ± 0.42; DCQD, 2.58 ± 0.52), intestine (SAP, 3.30 ± 0.68; DCQD, 2.50 ± 0.80) and lung (SAP, 3.30 ± 0.68; DCQD, 2.42 ± 0.52). DCQD reduced serum HMGB1 level (SAP, 134.09 ± 19.79; DCQD, 88.05 ± 9.19), RAGE level (SAP, 5.05 ± 1.44; DCQD, 2.13 ± 0.54). WB and RT-PCR showed HMGB1-RAGE pathway was inhibited by DCQD (*p* < 0.01).

**Discussion and conclusions:**

DCQD improves SAP-ALI in rats by interfering with intestinal lymphatic pathway and reducing HMGB1-induced inflammatory response.

## Introduction

Severe acute pancreatitis (SAP) is a disease with acute onset, rapid progression, several complications and high mortality. The mortality of systemic inflammatory response syndrome (SIRS) and multiple organ failure (MOF) caused by local haemorrhage and necrosis of the pancreas is as high as 20–40% (Boxhoorn et al. 2020). Among them, 65% of deaths are related to acute respiratory distress syndrome (ARDS), which is the final result of acute lung injury (ALI) (Mittal et al. 2011). Therefore, an in-depth study of the pathogenesis of SAP-ALI is of great significance in reducing mortality.

To date, the mechanism of SAP-ALI has not been fully explained. The role of intestinal barrier function is of great significance. Studies have found that 3/5 patients with SAP have intestinal barrier dysfunction (Wu et al. 2014). Moreover, the occurrence of SIRS, MOF and even death is closely related to intestinal barrier dysfunction in the early stages of SAP (Besselink et al. 2009; Ge et al. 2020). Intestinal barrier dysfunction leads to the translocation of intestinal flora and inflammatory factors. It can aggravate the inflammatory response, proving to be one of the key factors for the development of SIRS, multiple organ dysfunction syndrome (MODS), and even MOF, among which the most frequently affected are the lungs, cardiovascular system and kidney (Úbeda et al. 2016; Shanbhag et al. 2018; Zhang et al. 2020). However, the specific pathway remains to be explored.

Intestinal lymphatic pathway is an important pathway for the progression and deterioration of SAP. Inflammatory factors, bacteria, endotoxin and other harmful substances can be transferred from the intestine to other places through mesenteric lymph, resulting in SIRS, MODS and even death (Takeuchi et al. 2004; Úbeda et al. 2016; Boxhoorn et al. 2020; Zhang et al. 2020). Relevant animal studies have shown that mesenteric lymphatic drainage can reduce heart injury caused by acute pancreatitis (AP) in rats and improve the potential pathological oedema of the heart tissue (Shanbhag et al. 2018). Since the intestinal lymphatic pathway may be an important pathway for the development of SAP from a single organ to MODS or even MOF, our previous study discussed the pathogenesis of SAP-ALI based on the intestinal lymphatic pathway. It was found that by ligating the mesenteric lymph duct of SAP rats and intervening in the intestinal lymphatic pathway, the production of tumour necrosis factor alpha (TNF-α), interleukin (IL)-6, intercellular adhesion molecule-1 (ICAM-1) was significantly reduced, and the inflammatory injury induced by high mobility group box 1 (HMGB1) in lung tissues could be alleviated (Tang et al. 2021). Therefore, the role of the intestinal lymphatic pathway in SAP-ALI is further clarified. HMGB1, as an important inflammatory mediator, is worthy of attention.

Inflammatory factors are an important material basis for studying the pathogenesis of SAP-ALI. When SAP occurs, the changes in inflammatory factors IL-6 and TNF-α in the blood and mesenteric lymph nodes have been reported in details (Xiong et al. 2018; Fusco et al. 2020; Huang et al. 2020). However, the exact role of HMGB1 in tissue injury induced by AP and subsequent local or systemic inflammatory responses remains to further explore (Kang et al. 2014). Studies have found that HMGB1, as an inflammatory mediator, can stimulate the inflammatory cascade reaction and lead to SIRS, which is intimately associated in the intestinal lymphatic pathway and plays a crucial role in SAP-ALI (Christgen and Kanneganti 2021; Tang et al. 2021). This is the focus of our further study on the material basis of the intestinal lymphatic pathway in SAP-ALI.

To date, in addition to symptomatic treatment, there are no special methods or specific drugs to treat SAP-ALI (Yuan et al. 2017). Da-Cheng-Qi decoction (DCQD) characterized by ‘removing accumulation with purgation’ has a significant effect in the treatment of SAP-ALI (Zhao et al. 2011; Zhang et al. 2017; Yuan et al. 2018). DCQD first appeared in the Treatise on Febrile Diseases, one of the four classics of traditional Chinese medicine, in AD 200. Based on the intestinal barrier function and intestinal lymphatic pathway, this study discussed the effect of DCQD on HMGB1-induced inflammation in SAP-ALI rats to provide experimental data and theoretical support for clinical treatment.

## Materials and methods

### Animals

Forty-five male Sprague-Dawley (SD) rats (aged eight weeks, weighing 200 ± 20 g) were purchased from Shanghai Shrek Experimental Animal Co., Ltd., Chinese Academy of Sciences (license no: scxk [Shanghai] [2012-0002]). All rats were placed in a room (temperature: 22 °C; humidity: 55%) and adaptively fed for one week before the experiment. All procedures involving animals were carried out in accordance with the Guidelines for the Care and Use of Experimental Animals issued by the National Institutes of Health and approved by the Use and Management Committee of Experimental Animals of Yueyang Hospital of Integrated Traditional Chinese and Western Medicine affiliated to Shanghai University of Traditional Chinese Medicine (no. yylac-2018-087).

### Sodium taurocholate-induced SAP in rats

After fasting for 12 h, the SAP model was induced by a standard retrograde infusion of 5.0% sodium taurocholate solution (1 mg/kg) (Sigma, USA) at a constant rate of 12 mL/h using an infusion pump into the bile-pancreatic duct.

### Composition and preparation of DCQD

According to the original proportion of the Treatise on Febrile Diseases (4:8:4:3, g), DCQD consists of the following component herbs: the root of *Rheum officinale* Baill. (Polygonaceae) (no. 1705416) 12 g, the branch bark of *Magnolia officinalis* Rehder & E.H.Wilson (Magnoliaceae) (no. 1695809) 24 g, the fructus of *Citrus aurantium* L. (Rutaceae) (no. 1794375) 12 g and the crystal of *Natrii Sulfas* Glauber. (Sulfates) (no. 1675439) 9 g. The above traditional Chinese medicines are provided by the Traditional Chinese Medicine Pharmacy of Yueyang Hospital of Integrated Traditional Chinese and Western Medicine affiliated to Shanghai University of Traditional Chinese Medicine. The decocting method was performed in accordance with the Treatise on Febrile Diseases. Firstly, *M. officinalis* and *C. aurantium* were boiled in water 2000 mL for 1 h. Secondly, *R. officinale* was added and cooked for 5 min. Then *Natrii Sulfas* was melted in water. Finally, four layers of medical gauze were used to filter the solution. After centrifugation at 3000 rpm for 30 min at 25 °C, the crude drug was concentrated using a vacuum rotary evaporator at 45 °C to 1 g/mL. It was then stored at 4 °C and mixed well before use.

### Experimental design

**Figure 1.**
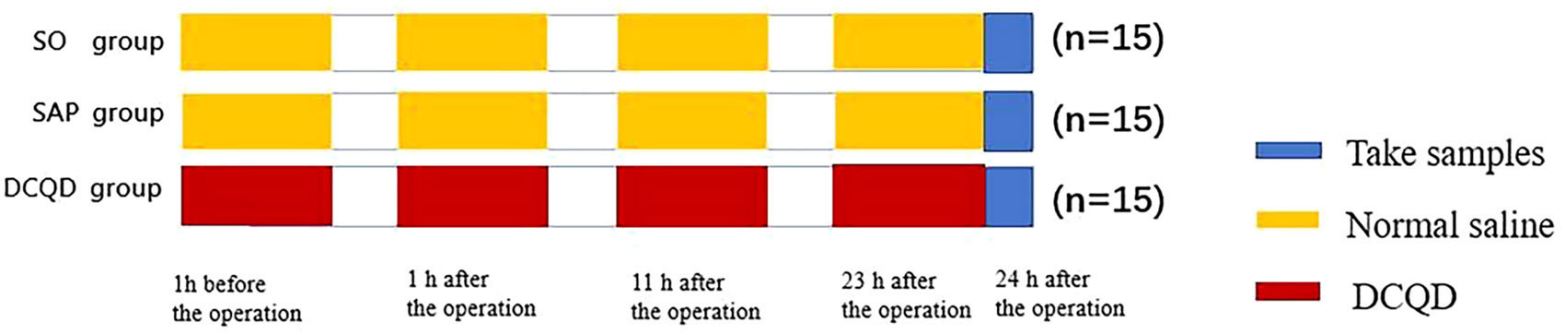
Experimental design. Forty-five Sprague-Dawley rats were divided into three groups: SO, SAP and DCQD groups (*n* = 15). The SAP model was induced by a standard retrograde infusion of 5.0% sodium taurocholate solution (1 mg/kg) at a constant rate of 12 mL/h using an infusion pump into the bile-pancreatic duct. The SO and SAP group were given 0.9% normal saline, while DCQD group was given DCQD (5.99 g/kg/d) by gavage 1 h before operation and 1 h, 11 h and 23 h after operation. 24 h after operation, samples were taken for detection.

Forty-five SD rats were randomly divided into three groups (Figure 1).

Sham operation group (SO group) (*n* = 15): After disinfection, open the abdomen, turn over the pancreas and close the abdomen. Normal saline (0.45 mL each time) was administered 1 h before the operation and 1, 11 and 23 h after the operation.

SAP group (*n* = 15): The modelling method was the same as above, and normal saline (0.45 mL each time) was administered 1 h before operation and 1, 11 and 23 h after the operation.

DCQD group (*n* = 15): The modelling method was the same as above, and DCQD (5.99 g/kg/d) was administered by gavage 1 h before the operation and 1, 11 and 23 h after the operation.

After 24 h, 2.0% pentobarbital sodium (Sinopharm Chemical Reagent Co., Ltd., China) was injected intraperitoneally for anaesthesia and blood samples were obtained from the abdominal aorta, followed by centrifugation at 1200 *g* for 15 min at 4 °C. The serum was cryopreserved at −80 °C. In addition, pancreas, lung, ileum tissue and terminal ileum were collected for testing.

### Analysis of pathological specimens

Pancreatic heads and left lower lung of rats were fixed with 4% paraformaldehyde solution, embedded in paraffin, and the sections were stained with haematoxylin-eosin (H&E) solution. According to the scoring standard methods, the pathological scores were calculated (Imanaka et al. 2001; Kubisch et al. 2004).

### Observation of intestinal tissue structure under electron microscope

The fixed intestinal tissue specimens were washed with phosphate-buffered saline (PBS), fixed with 1% osmium tetroxide solution, dehydrated, embedded and then stained with uranyl acetate and lead citrate. The intestinal microvilli and epithelial cell junction structures were observed under a fluoroscopic electron microscope (Bar = 2 µm).

### Detection of myeloperoxidase (MPO) levels

Lung tissue samples were thawed, homogenized in 20 mM phosphate-buffered saline (PBS) (pH = 7.4) and centrifuged at 1200 *g* for 20 min at 4 °C. The supernatant was assayed for MPO activity using enzyme-linked immunosorbent assay (ELISA) kits (Nanjing Jiancheng, China). All procedures were conducted according to the manufacturer’s instructions.

### Measurement of biochemical indexes in serum

According to the instructions of the commercially available ELISA kit (Nanjing Jiancheng, China), measure the content of HMGB1, RAGE, TNF-α, IL-6, ICAM1, DAO and d-LA (λ = 450 nm).

### Real‑time polymerase chain reaction (RT‑PCR)

According to the instructions of the TRIzol kit (Invitrogen, USA), extract the total RNA and carry out cDNA amplification (Takara, JPN). Fluorescence quantitative PCR was performed with SYBR Green PCR Master MIX kit. (Applied Biosystems, USA). The primers were synthesized by Sangon Biotech (Shanghai, China). The gene primer sequences are shown in Table 1.

**Table 1.** Primer sequences of gene.

Gene	5′-3′	3′-5′
HMGB1	CGCGGAGGAAAATCAACTAA	GGGTGCTTCTTCTTGTGCTC
RAGE	AGGAACGTGCAGAGCTGAAT	AGAAAGTGGCTCGAGGTTGA
NF-κB p65	GCGTTTCCGTTACAAGTGCG	GTGCGTCTTAGTGGTATCTGTGC
TNF-α	TGCCTCAGCCTCTTCTCATT	CCCATTTGGGAACTTCTCCT
β-actin	AGCCATGTACGTAGCCATCC	ACCCTCATAGATGGGCACAG

### Western blotting analysis

The protein lysate was used to extract lung tissue and mesenteric lymph node protein and determine the concentration. After sodium dodecyl sulphate-polyacrylamide gel electrophoresis, the protein was transferred to the polyvinylidene fluoride membrane by electrophoresis. The membranes were blocked with 5% skimmed milk and then incubated with primary antibodies (HMGB1 1:1000, RAGE 1:800, NF-kB p65 1:1000) (Cambridge, UK; Ab18256, Ab3611, Ab16502) and (GAPDH 1:1500, H3 1:1500) (Danvers, MA, USA, #5471, #8173) overnight at 4 °C. The membranes were incubated with a horseradish peroxidase-conjugated anti-rabbit antibody (1:1000, Beyotime, Shanghai, China, Ab18256) at 25 °C for 1 h. After the membrane is washed with eluent, the immunoreactive bands were visualized using ECL Plus (Life Technology Corporation, Gaithersburg, MD, USA) with a ChemiDoc XRS bioimaging system (Bio-Rad Laboratories, Inc., Hercules, CA, USA).

### Statistical analysis

Data are presented as mean ± standard deviation (SD). Statistical analyses were conducted with GraphPad Prism 7.0 software. The statistical tests one-way ANOVA analysis and the least significant difference (LSD) test were performed to observe the significant differences between the sample means. *p* < 0.05 was considered statistically significant.

## Results

### Changes of pancreatic tissues in each group

**Figure 2.**
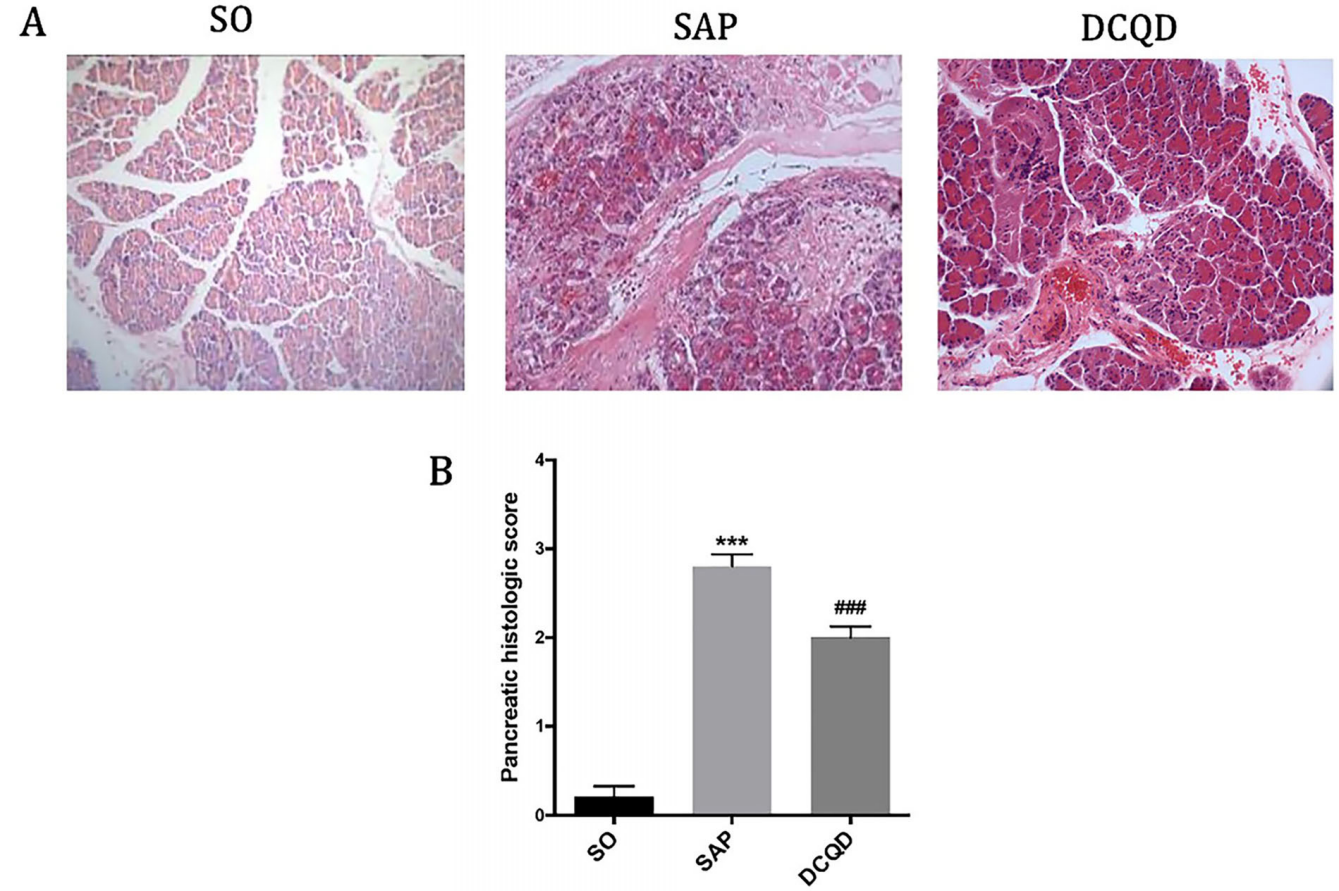
Impact of DCQD on STC-induced pancreatic injury. (A) Images of H&E staining, 200 × magnifications of pancreas tissues of each experimental group. (B) Pathological score of pancreas tissues of each experimental group. ***p < 0.001, compared with the SO group; ^###^*p* < 0.001, compared with the SAP group. *n* = 10–14.

As shown in Figure 2(A), In the SAP group, the pancreatic tissue showed coagulative necrosis, mixed with large haemorrhagic foci, interstitial oedema and a large amount of inflammatory cell infiltration compare with the SO group. In the DCQD group, pancreatic congestion and oedema were mild, and inflammatory cell infiltration was less. Figure 2(B) shows the pancreatic pathological scores of the different groups. The pancreatic pathological scores in the SAP group were significantly higher than those in the SO group (2.80 ± 0.42 *vs.* 0.29 ± 0.47; *p* < 0.001). The pancreatic pathological scores in the DCQD group were significantly lower than those in the SAP group (2.58 ± 0.52 *vs.* 2.80 ± 0.42; *p* < 0.001).

### Changes of lung tissues in each group

**Figure 3.**
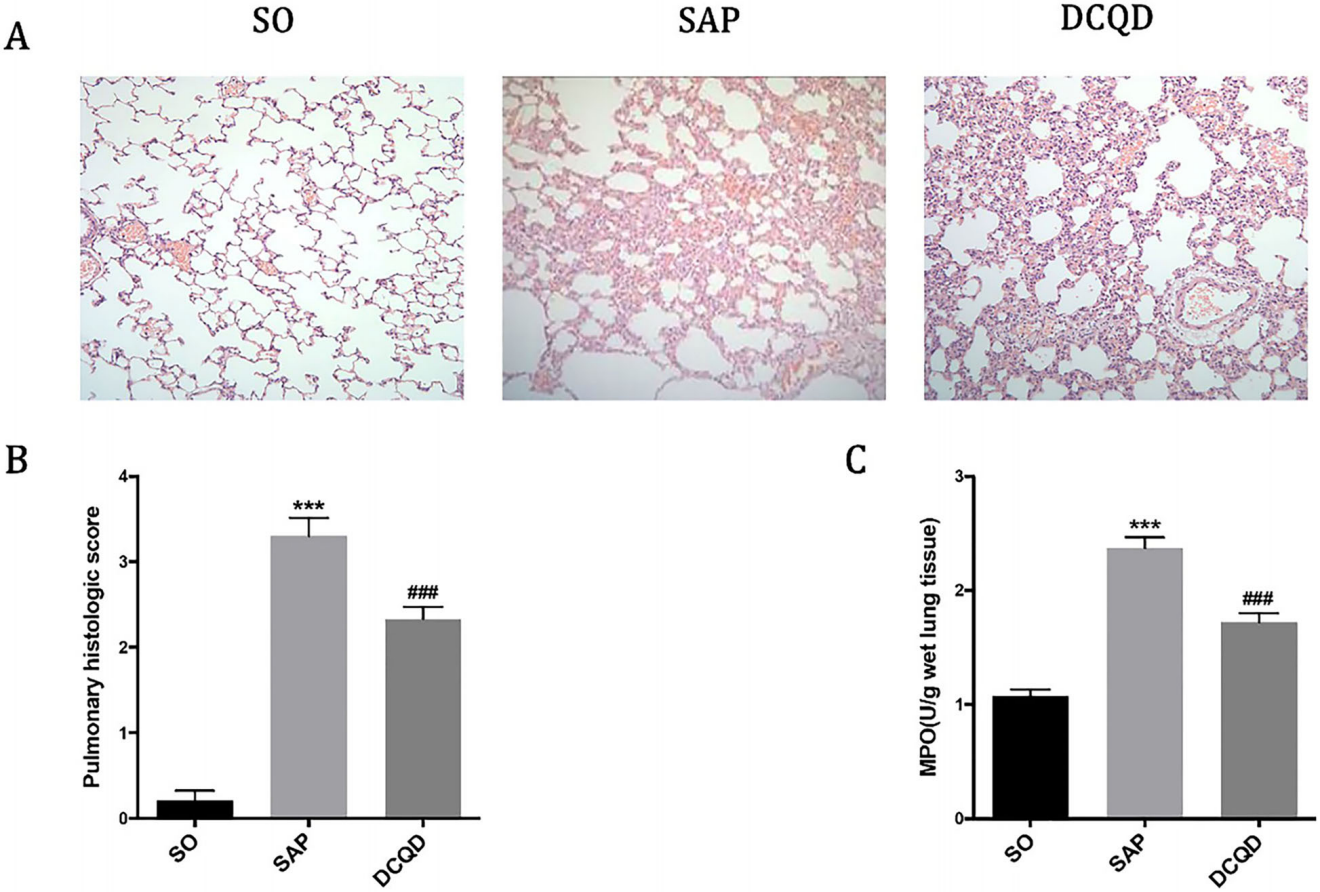
Impact of DCQD on SAP-associated lung injury. (A) Images of H&E staining, 200 × magnifications of lung tissues of each experimental group. (B) Pathological score of lung tissues of each experimental group. (C) MPO activity in lung tissues of each experimental group. ***p < 0.001, compared with the SO group; ^###^*p* < 0.001, compared with the SAP group. *n* = 10–14.

As shown in Figure 3(A), the lung tissue structure of rats in the SO group was normal, and no abnormal histological changes were observed under light microscope. The histopathological changes in the lungs in the SAP group mainly showed extensive thickening of the alveolar wall caused by pulmonary oedema, alveolar congestion, alveolar collapse and inflammatory cell infiltration. Compared with the SAP group, interstitial oedema and inflammatory cell infiltration of lung tissue in the DCQD group were significantly reduced. According to the pathological score of lung tissue (Figure 3(B)), the lung pathological score of the SAP group was significantly higher than that of the SO group (3.30 ± 0.68 *vs.* 0.29 ± 0.47; *p* < 0.001). The pulmonary pathological score was significantly lower in the DCQD group than that in the SAP group (2.42 ± 0.52 *vs.* 3.30 ± 0.68; *p* < 0.001).

In addition, we measured the MPO activity in lung tissue to assess the degree of neutrophil infiltration and the severity of inflammation in the lungs. As shown in Figure 3(C), the MPO activity in the lungs of the SO group was lower and significantly increased 24 h after the induction of SAP (1.08 ± 0.20 *vs.* 2.37 ± 0.30; *p* < 0.001), whereas the MPO activity of rats treated with DCQD (1.72 ± 0.27) by gavage decreased significantly (*p* < 0.001).

### Changes of intestinal tissues in each group

**Figure 4.**
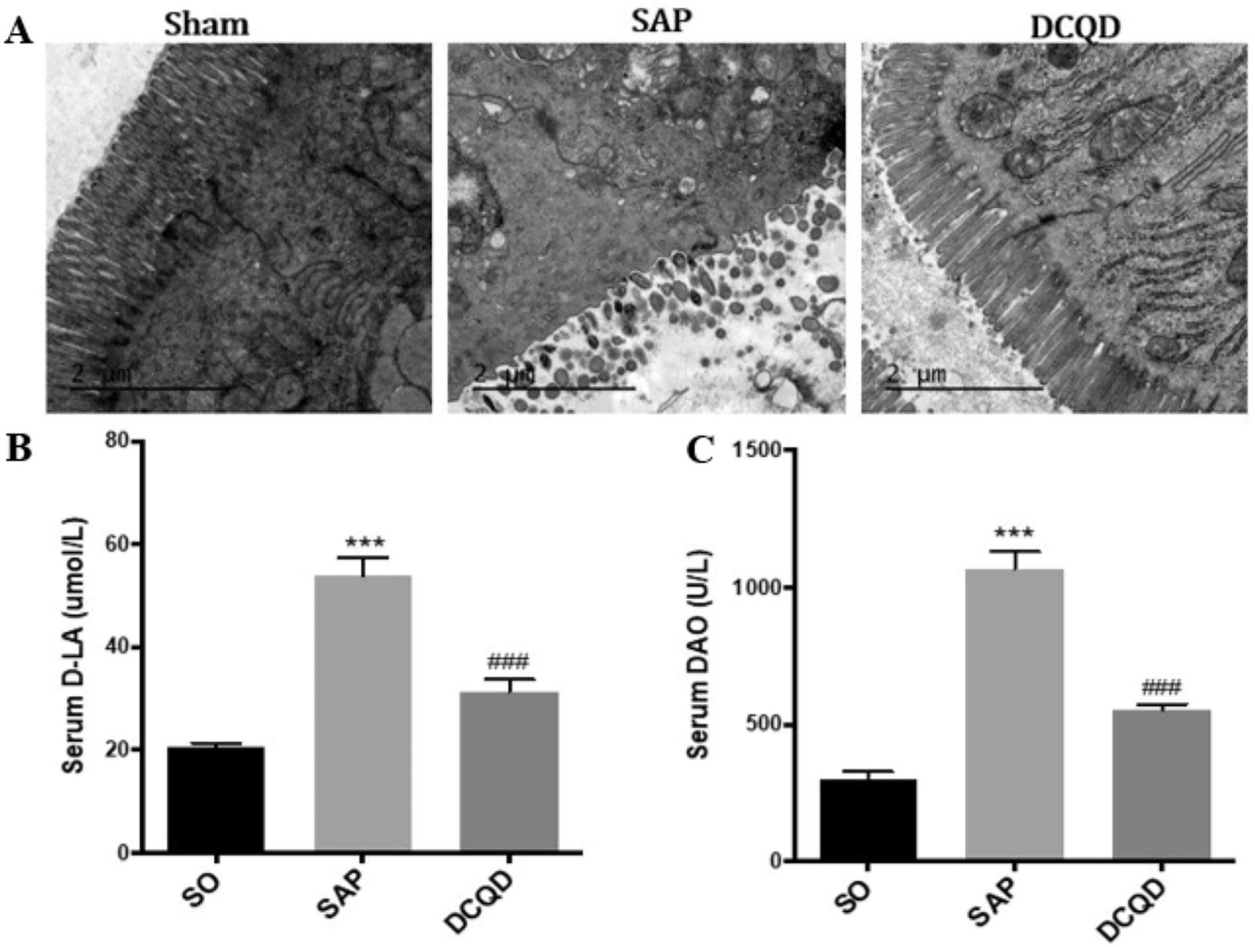
Impact of DCQD on SAP-associated intestinal injury. (A) Electron microscopic images of intestinal tissue of each experimental group (2 µm). (B) Serum d-LA levels of each experimental group. (C) Serum DAO levels of each experimental group. ***p < 0.001, compared with the SO group; ^###^*p* < 0.001, compared with the SAP group. *n* = 10–14.

As shown in Figure 4(A), electron microscopy revealed that the SO group has complete intestinal epithelial villi, normal organelle morphology and intact tight junctions. However, the SAP group had significantly damaged intestinal mucosa, sparse exfoliated intestinal epithelial villi and widened intercellular space. Moreover, after DCQD, there was no significant shedding of epithelial cells from the intestinal villi of rats, the cells were closely connected, and the mitochondria did not swell significantly. In addition, we also tested the levels of d-LA and DAO to assess intestinal damage and found that the levels of serum d-LA and DAO were significantly higher in the SAP group (d-LA, 53.97 ± 10.32; DAO, 1068.90 ± 200.47) than in the SO group (d-LA, 20.46 ± 3.45; DAO, 296.83 ± 109.64; *p* < 0.001) and the levels of d-LA and DAO were significantly lower in the DCQD group (d-LA, 31.45 ± 7.78; DAO, 551.52 ± 74.65) than in the SAP group (*p* < 0.001; Figure 4(B,C)).

### The expression levels of HMGB1, RAGE and NF‑κB p65 in lung tissues

**Figure 5.**
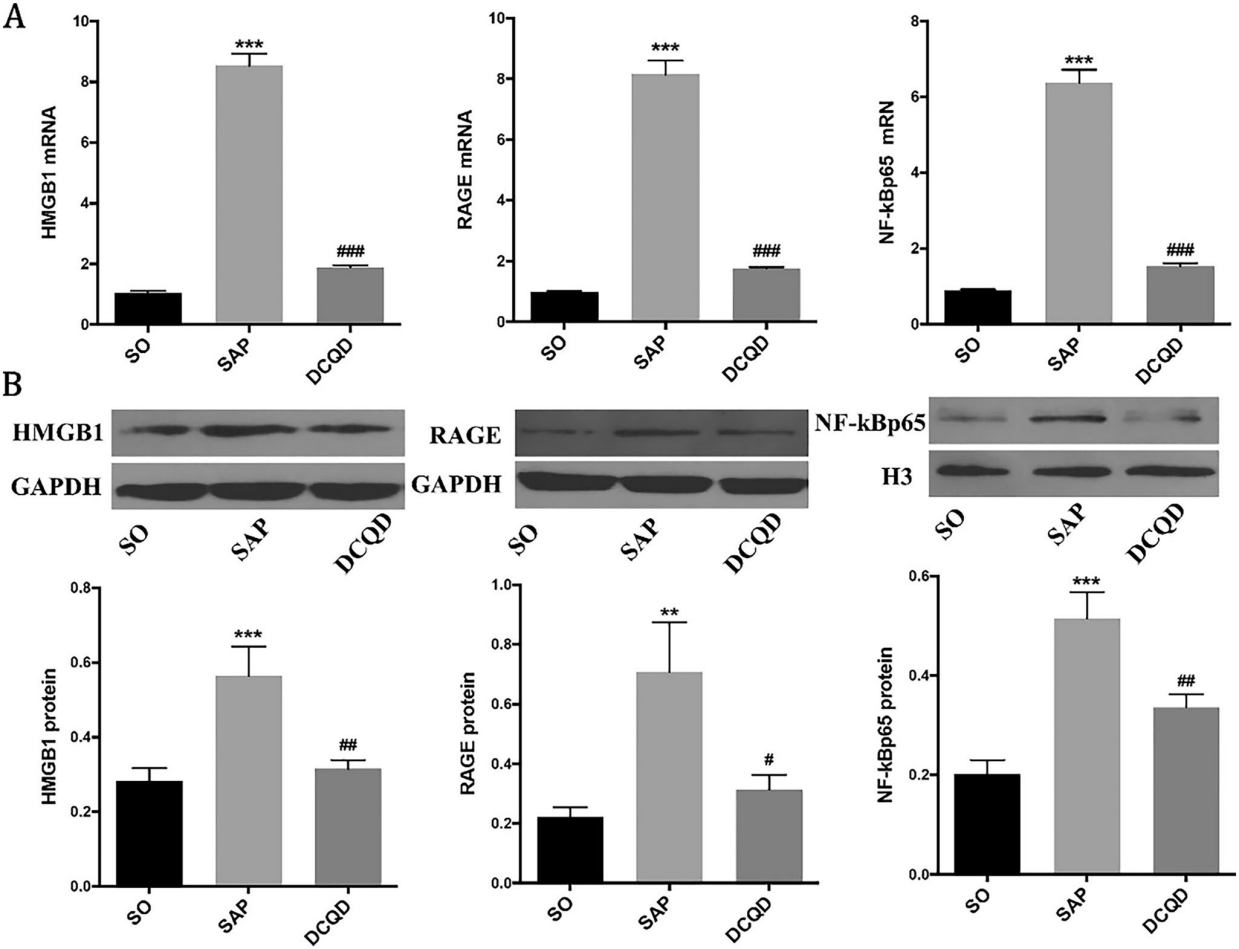
Impact of DCQD on the expression of HMGB1, RAGE and NF-κB p65 at the mRNA and protein levels in lung tissues. (A) RT-PCR analysis of HMGB1, RAGE and NF-κB p65 mRNA expression in lung tissues. (B) Western blot results of HMGB1, RAGE and NF-κB p65 protein expression in lung tissues. (C) Statistical results of B scanning densitometry. **p < 0.01, ***p < 0.001, compared with the SO group; ^#^*p* < 0.05, ^##^*p* < 0.01, ^###^*p* < 0.001, compared with the SAP group. *n* = 10–14.

We used RT-PCR and Western blot analysis to investigate whether DCQD can affect the expression of HMGB1 and its downstream signals in the lungs (Figure 5(A,B)). The results showed that the activity of HMGB1 in the lung tissue of SAP rats increased, whereas the use of DCQD decreased its activity (8.53 ± 1.26 *vs.* 1.88 ± 0.21; *p* < 0.001). Meanwhile, the expression of RAGE mRNA in the lung tissue of SAP rats also increased significantly, and intragastric administration of DCQD effectively inhibited this increase (8.15 ± 1.76 *vs.* 1.76 ± 0.17; *p* < 0.001). We also found that the expression of NF-κB p65 in SAP rats significantly increased at the mRNA level, whereas DCQD inhibited this upregulation (6.38 ± 1.06 *vs.* 1.52 ± 0.28; *p* < 0.001; Figure 5(A)).

In addition, Western blot analysis showed that the expression of HMGB1 and NF-κB p65 in the SAP group (HMGB1, 0.56 ± 0.25; NF-κB p65, 5.05 ± 1.44) was significantly increased compared with that in the SO group (HMGB1, 0.28 ± 0.13; NF-κB p65, 1.58 ± 0.32; *p* < 0.001). However, they all distinctly decreased in the DCQD group (HMGB1, 0.36 ± 0.11; NF-κB p65, 2.13 ± 0.54) compared with the SAP group (*p* < 0.01). Western blot analysis also showed that the expression of RAGE in the SAP group (0.70 ± 0.53) increased compared with that in the SO group (0.22 ± 0.12; *p* < 0.01) and decreased in the DCQD group (0.42 ± 0.32) compared with that in the SAP group (*p* < 0.05; Figure 5(B)).

### The expression levels of HMGB1, RAGE, TNF-α, IL-6 and ICAM1 in serum

**Figure 6.**
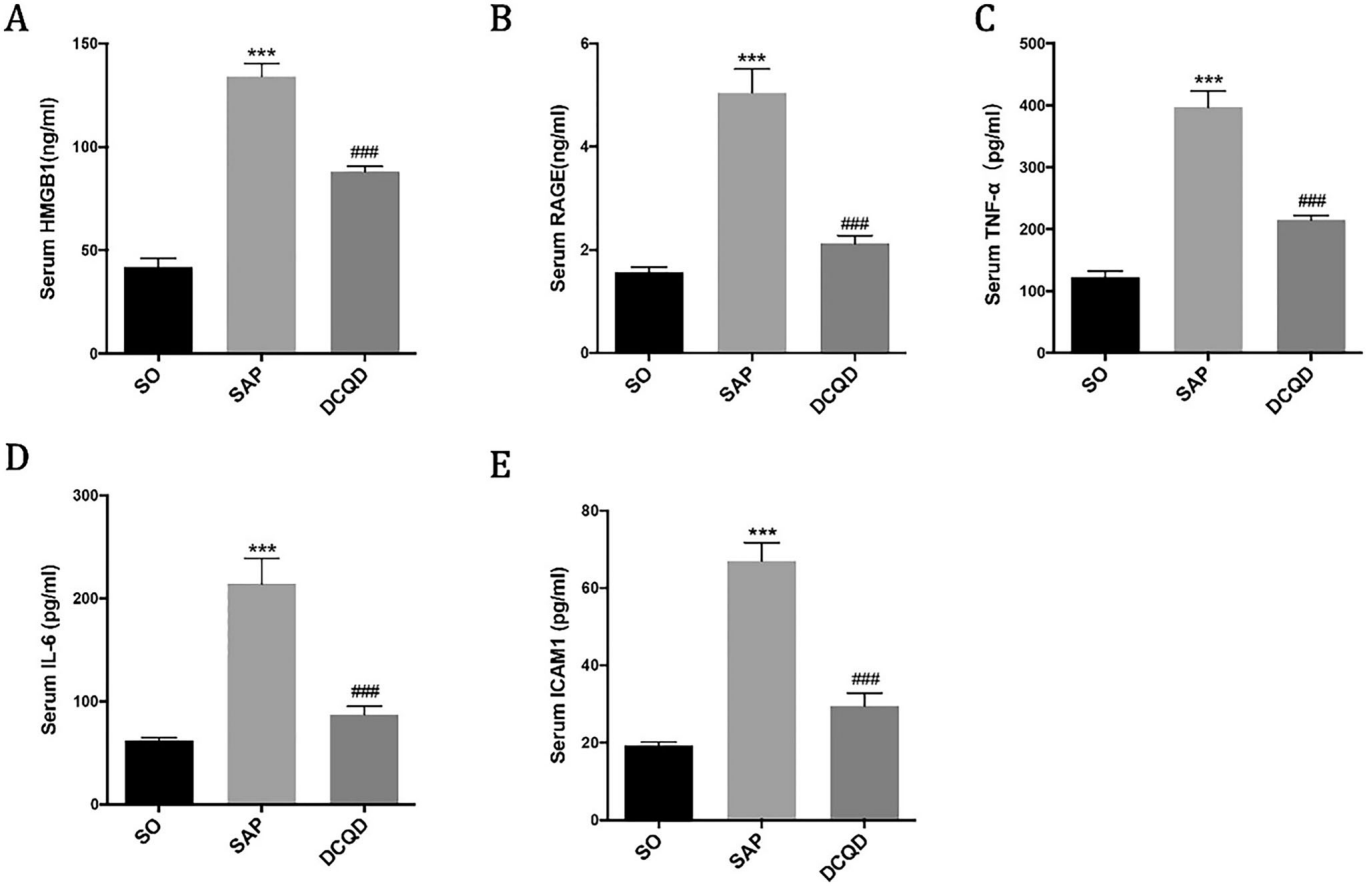
Impact of DCQD on the levels of inflammatory factors in serum. ELISA data of HMGB1, RAGE, TNF-α, IL-6 and ICAM1 in serum. ***p < 0.001, compared with the SO group; ^###^*p* < 0.001, compared with the SAP group. *n* = 10–14.

We used ELISA to determine the concentrations of HMGB1 (Figure 6(A)), RAGE (Figure 6(B)), TNF-α (Figure 6(C)), IL-6 (Figure 6(D)) and ICAM1 (Figure 6(E)) in serum. The results showed that the serum levels of HMGB1, RAGE, TNF-α, IL-6 and ICAM1 in the SAP group (HMGB1, 134.09 ± 19.79; RAGE, 5.05 ± 1.44; TNF-α, 396.88 ± 82.67; IL-6, 214.19 ± 77.57; ICAM1, 5.05 ± 1.44) were significantly higher than those in the SO group (HMGB1, 42.00 ± 15.72; RAGE, 1.58 ± 0.32; TNF-α, 122.34 ± 39; IL-6, 62.17 ± 10.05; ICAM1, 1.58 ± 0.32; *p* < 0.001). Compared with the SAP group, the serum levels of HMGB1, RAGE, TNF-α, IL-6 and ICAM1 in the DCQD group (HMGB1, 88.05 ± 9.19; RAGE, 2.13 ± 0.54; TNF-α, 214.58 ± 26.46; IL-6, 87.34 ± 27.66; ICAM1, 2.13 ± 0.54) significantly decreased (*p* < 0.001).

### The mRNA expression levels of HMGB1, RAGE and TNF-α in mesenteric lymph node

**Figure 7.**
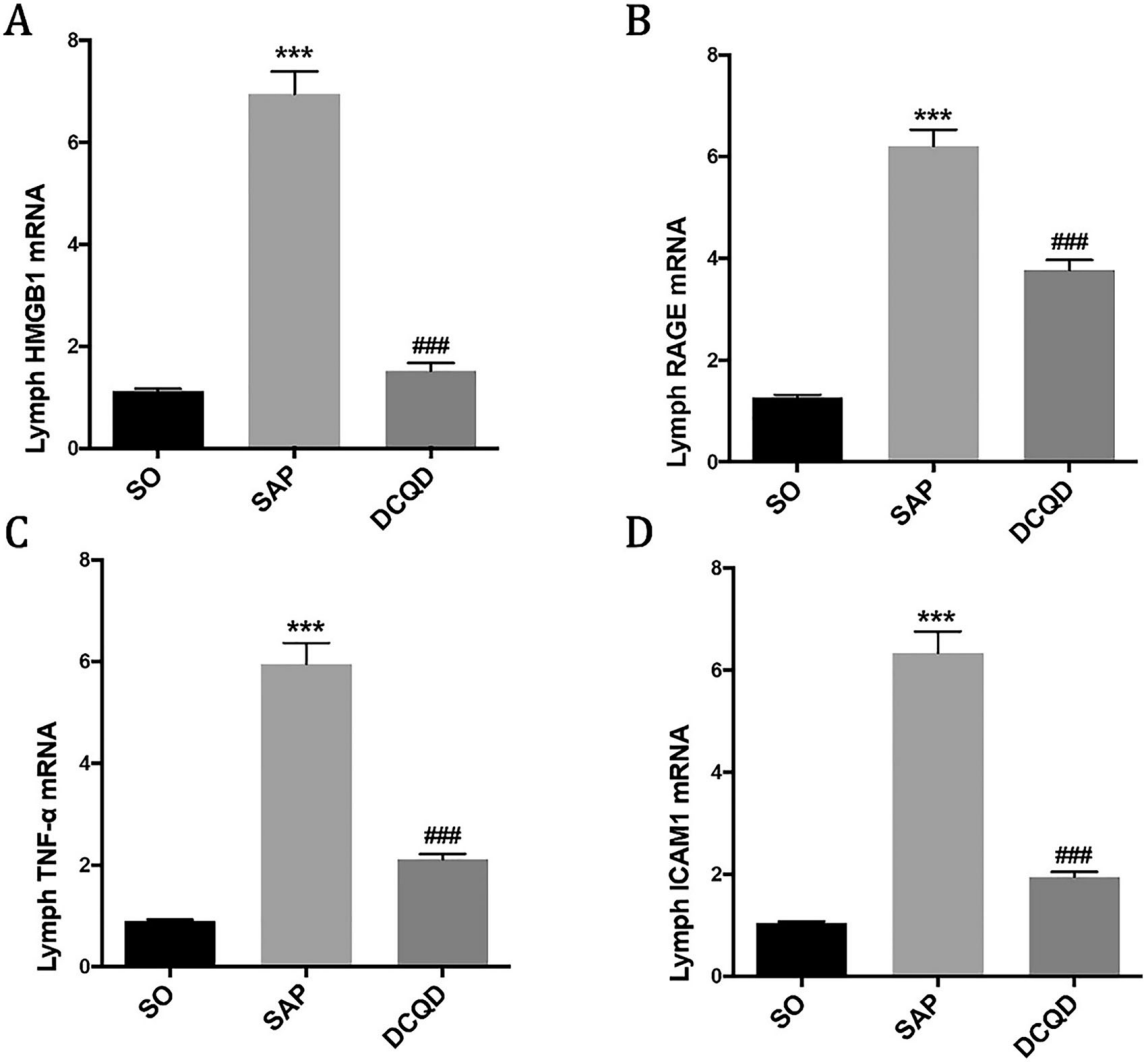
Impact of DCQD on the levels of inflammatory factors in mesenteric lymph node. RT-PCR analysis of HMGB1, RAGE, TNF-α and ICAM1 mRNA in mesenteric lymph node. ***p < 0.001, compared with the SO group; ^###^*p* < 0.001, compared with the SAP group. *n* = 10–14.

Our experiment explored whether the DCQD intervention could affect the mRNA levels of HMGB1 (Figure 7(A)), RAGE (Figure 7(B)), TNF-α (Figure 7(C)) and ICAM1 (Figure 7(D)) in mesenteric lymph node. The results showed that the mRNA levels of HMGB1, RAGE, TNF-α and ICAM1 in mesenteric lymph node in the SAP group (HMGB1, 6.95 ± 1.39; RAGE, 7.53 ± 1.18; TNF-α, 5.95 ± 1.31; ICAM1, 6.35 ± 1.32) were significantly higher than those in the SO group (HMGB1, 1.13 ± 0.15; RAGE, 1.26 ± 0.21; TNF-α, 0.90 ± 0.11; ICAM1, 1.04 ± 0.13; *p* < 0.001), and the mRNA levels of HMGB1, RAGE, TNF-α and ICAM1 in the mesenteric lymph node of the DCQD group (HMGB1, 1.52 ± 0.54; RAGE, 2.08 ± 0.34; TNF-α, 2.11 ± 0.36; ICAM1, 1.95 ± 0.32) were significantly lower than those in the SAP group (*p* < 0.001).

## Discussion

Traditional Chinese medicine, especially DCQD, characterized by ‘removing accumulation with purgation’, has a definite clinical curative effect on SAP, which has gradually become a focus of attention. However, the specific mechanisms involved are not clear. It has been found that the method of ‘removing accumulation with purgation’ can effectively promote gastrointestinal motility; regulate intestinal endothelial barrier function-related proteins AQP-1, MMP9 and JAM-C; improve intestinal barrier dysfunction; inhibit intestinal bacteria and endotoxin translocation; regulate inflammatory response; and regulate the PI3K/Akt signalling pathway or JAK2/STAT3 signalling pathway to reduce the severity of SAP (Zhao et al. 2015; Pan et al. 2017; Yuan et al. 2018; Jin and Shen 2019; Su et al. 2019; Sun et al. 2020). However, further research is needed to explore the specific mechanism of DCQD in the treatment of SAP-ALI based on the inflammatory response.

In this study, we successfully established an SAP rat model through retrograde injection of 5% sodium taurocholate into the biliopancreatic duct. Based on the intestinal barrier function and intestinal lymphatic pathway, we discussed the effect of DCQD on HMGB1-induced inflammatory response to explore the therapeutic mechanism of DCQD on SAP-ALI. Our results showed that DCQD could improve the intestinal barrier function by reducing inflammatory factors such as HMGB1 in the intestine, reduce their translocation through the intestinal lymphatic pathway and reduce the inflammatory response induced by HMGB1, which plays an important role in the treatment of SAP-ALI.

The intestinal mucosa is mainly composed of mechanical, immune, chemical and biological barriers. It plays an important role in the maintenance of intestinal function and pathogenesis of AP (Huang et al. 2019; Zhang et al. 2021). Damage to intestinal epithelial cells, host immune deficiency and disturbance of intestinal flora can lead to increased intestinal permeability, which are the three key factors leading to intestinal barrier dysfunction and bacterial and endotoxin translocation (Pan et al. 2017). When the intestinal barrier function is damaged, a large number of endotoxins, inflammatory factors and harmful substances produced in the intestinal cavity can migrate to the lungs, damage the pulmonary vascular endothelial cells and alveolar epithelial cells and destroy the pulmonary blood-gas barrier. With an increase in pulmonary vascular permeability, protein-rich fluid spills into the alveoli and lung interstitium, causing pulmonary oedema and diffuse alveolar damage. Finally, ALI characterized by hypoxaemia appeared (Besselink et al. 2009; Ge et al. 2020; Li et al. 2020; Shi et al. 2021).

d-LA and DAO are two specific indicators that reflect the intestinal barrier function (Song et al. 2009; Cai et al. 2019). d-LA is a metabolite of the inherent bacteria in the gastrointestinal tract. When the body suffers from trauma, shock, acute intestinal ischaemia, intestinal obstruction, or other causes of intestinal barrier function damage, resulting in an increase in intestinal permeability, a large amount of d-LA produced by intestinal bacteria enters the blood circulation through the damaged intestinal mucosa, resulting in an increase in blood d-LA levels; therefore, serum d-LA content in peripheral blood can be used as an important indicator to monitor intestinal permeability and intestinal barrier function damage (Chen et al. 2018; Yang et al. 2021). DAO is an intracellular enzyme that catalyses diamine in the mammalian intestinal mucosa or ciliated epithelial cells. Its activity in blood is stable, and it can protect the intestinal mucosa by regulating ion balance in cells, affecting the conduction pathway and promoting cell repair. The damaged mucosal cells release DAO and then increase its serum concentration; therefore, the concentration of DAO in the blood can reflect the damage and recovery of the intestinal cavity in a timely manner (Kong et al. 2015; Fusco et al. 2020). In clinical and experimental studies, serum d-LA and DAO levels are usually measured to evaluate the damage to intestinal cells and intestinal barrier function. Our results showed that the levels of d-LA and DAO in the serum significantly increased after modelling. Electron microscopy showed that the intestinal mucosa of SAP rats was damaged obviously. However, the levels of DAO and d-LA decreased significantly in the DCQD group, and damage to the intestinal mucosa ultrastructure was significantly reduced under an electron microscope. These results showed that DCQD can reduce intestinal injury, improve intestinal barrier function and have a certain therapeutic effect on SAP-ALI. However, the specific path needs to be further discussed.

To date, there are several different views on the translocation pathways of harmful substances such as bacteria and endotoxins in AP, including haematogenous, transperitoneal, lymphogenic, colonic transmural and pancreatic duct translocations (Liu et al. 2019). The exact pathway has not been fully elucidated, but impairment of intestinal barrier function is a common feature of all potential pathways. It is believed that there are two main routes for bacterial endotoxins or toxic substances produced by inflammatory reactions to break through the intestinal barrier and migrate to the circulation: the portal vein and intestinal lymphatic pathways. The portal vein pathway refers to the circulation of bacteria and endotoxins or other substances through the mesenteric venules, portal vein and liver to the systemic circulation (Qin et al. 2002). The intestinal lymphatic pathway refers to the return of bacteria, endotoxin, or other substances to the cisterna chyli through the lymph nodes and lymphatic vessels of the intestinal lymphatic system, which then enters the blood through the thoracic duct (Escobedo and Oliver 2017). The traditional theory of intestinal barrier dysfunction and enterogenic infection states that displaced bacteria and endotoxins return to the liver through the portal system and spread to the whole body (Lichtman 2001). However, some studies found that bacteria and endotoxins were not detected in the portal vein blood and peripheral blood of patients with MOF (He et al. 2011).

It should be noted that lymphatic ligation and drainage can prevent organ dysfunction and improve pulmonary neutrophil aggregation in animals with haemorrhagic shock. In addition, pathological lung injury caused by haemorrhagic shock can be reproduced by injecting the lymph of haemorrhagic shock animals into normal animals (Senthil et al. 2007). Therefore, we speculate that the gut-derived factors leading to distant organ injury may not directly enter the systemic circulation but may cause an immune response in local gut-associated lymphoid tissue or draining lymph nodes, resulting in a significant systemic response. Intestinal barrier dysfunction in SAP leads to the occurrence of ALI, which may be closely related to the entry of toxic substances from the intestinal lymphatic system through the thoracic duct into the lung (Deitch 2012; Schietroma et al. 2018; Haussner et al. 2019). Anatomically, the lung is the first organ to receive enterogenous toxic substances mediated by intestinal lymph nodes, which can explain why the lung is the first organ to be involved in clinical trauma and shock (Reed et al. 2019).

In order to explore the role of intestinal lymphatic pathway in SAP-ALI, the first half of our experiments found that SAP-ALI could be alleviated by ligating the intestinal lymph of SAP rats, suggesting that intestinal bacterial endotoxins or toxic substances produced by intestinal inflammatory responses can entering the lungs *via* the intestinal lymphatic pathway, and ligation of the mesenteric lymphatic vessels can block the primary pathway (Tang et al. 2021). However, we also found that mesenteric lymphatic ligation may lead to the accumulation of a large number of bacteria and toxins in the intestine, resulting in serious intestinal injury. In this study, we found that the intestinal tissue injury was improved by DCQD compared with that in the model group, which suggests that DCQD can also treat SAP-ALI and reduce the damage caused by inflammatory mediators or toxic substances entering the lung through the intestinal lymphatic pathway. However, different from mesenteric lymphatic ligation, DCQD can reduce the intestinal damage caused by excessive accumulation of harmful substances in the intestine, improve the intestinal barrier function and reduce ALI caused by inflammatory mediators or toxic substances entering the lungs through the intestinal lymphatic pathway. Among them, inflammatory mediators play an important role in the pathological process, which is also the focus of our study.

Current studies have found that SAP-ALI is closely related to the production of inflammatory factors. There have been many studies on inflammatory mediators such as TNF-α and IL-6 (Úbeda et al. 2016; Su et al. 2019; Ge et al. 2020). However, to the best of our knowledge, the roles of HMGB1 and RAGE in SAP-ALI have not been fully elucidated yet. HMGB1 is a highly conserved protein that exists in the nucleus and cytoplasm of almost all cell types. The expression of HMGB1 is increased later and appears after the ‘first hit’, but it lasts for a long time (Xu et al. 2014; Yang et al. 2020). Studies have found that HMGB1 can mediate a severe inflammatory response. When cells are in a state of stress, HMGB1 is released from the nucleus to the outside of cells which promotes monocytes and macrophages to secrete pro-inflammatory factors. In this way, a positive feedback loop is established to amplify the inflammatory response, produce a cascade effect, aggravate the systemic inflammatory response and finally develop SIRS or sepsis (Chen et al. 2015; Kim et al. 2021). Furthermore, the inflammatory function of HMGB1 is mediated by its binding to cell surface receptors, and RAGE is one of its main receptors (Yao and Brownlee 2010; Tang et al. 2012). RAGE is a receptor of the Toll-like receptor superfamily and a type I transmembrane protein, which comprises two cytoplasmic domains and a variable extracellular domain. It is considered to be a receptor mediating HMGB1 chemotaxis and cytokine activity in macrophages and tumour cells and plays an important role in inflammation and autoimmunity (Guarneri et al. 2021). As demonstrated by our data, the impairment of intestinal barrier function leads to the transfer of intestinal inflammatory factors such as HMGB1, which combines with RAGE to activate NF-κB pathway and exacerbates inflammation. The activation of NF-κB pathway is an important factor to damage in many organs, and the p65 subunit is a major pro-inflammatory subunit whose activation plays a crucial role in the pathogenesis of SAP. The combination of HMGB1 and RAGE can promote intracellular signal transduction, such as the NF-κB pathway, which triggers the release of various cytokines, such as TNF-α and IL-6, leading to SIRS, MOD and even death (Kang et al. 2014; Jabaudon et al. 2015; Tang et al. 2021). Our study found that compared with SAP rats, the serum levels of HMGB1, RAGE, TNF-α, IL-6 and ICAM1 in the DCQD group were significantly reduced. Similarly, after DCQD administration, the mRNA levels of HMGB1, RAGE, TNF-α and ICAM1 in mesenteric lymph nodes and the expression of HMGB1, RAGE and NF-κB p65 at mRNA and protein levels in lung tissues were significantly decreased. These results suggest that the inflammatory response induced by HMGB1 aggravates SAP-ALI, whereas DCQD reduces the translocation of HMGB1 to the lung through the intestinal lymphatic pathway, reduces the binding with RAGE, inhibits the NF-κB p65 signalling pathway and reduces the release of various cytokines, such as TNF-α, IL-1β and IL-6, which plays an important role in the treatment of SAP-ALI.

## Conclusions

**Figure 8.**
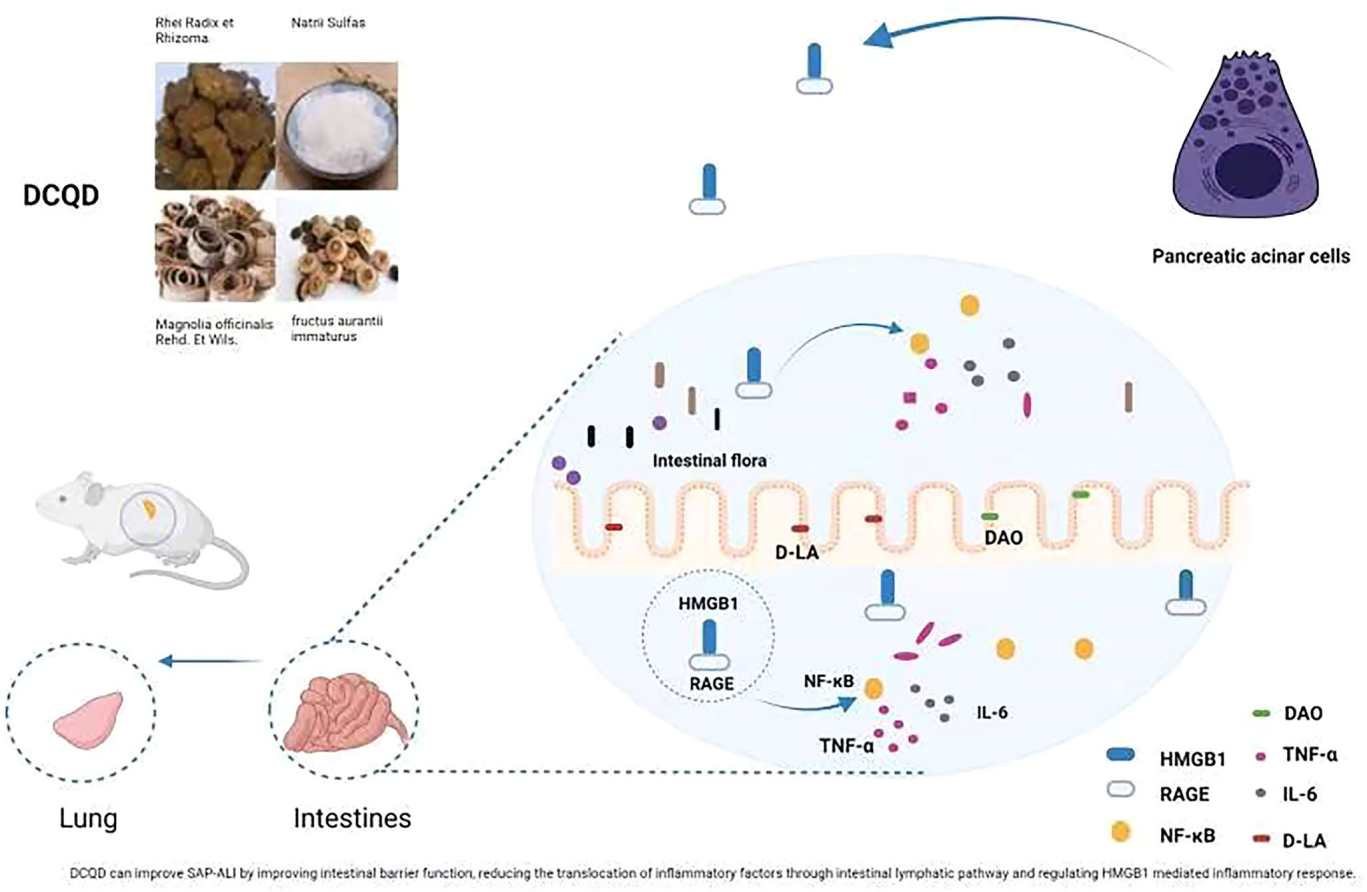
DCQD can improve SAP-ALI by improving intestinal barrier function, reducing the translocation of inflammatory factors through intestinal lymphatic pathway and regulating HMGB1 mediated inflammatory response. DCQD: Da-Cheng-Qi decoction; SAP-ALI: severe acute pancreatitis-associated acute lung injury; HMGB1: high mobility group box 1. RAGE: receptor of advanced glycation end products; NF-κB: nuclear factor kappa-B; TNF-α: tumour necrosis factor alpha; DAO: diamine oxidase; d-LA: d-lactate.

Our study demonstrated that DCQD could protect the intestinal barrier by reducing the production of inflammatory factors such as HMGB1, RAGE and TNF-α in the intestine; it could reduce their translocation through the intestinal lymphatic pathway and inhibit the inflammatory response induced by HMGB1 to treat SAP-ALI (Figure 8). Hence, DCQD could be a promising approach for the treatment of SAP-ALI.
